# Development, Implementation, and Evaluation of a Distance Learning and Telementoring Program for Cervical Cancer Prevention in Cameroon

**DOI:** 10.1001/jamanetworkopen.2022.40801

**Published:** 2022-11-08

**Authors:** Joel Fokom Domgue, Mala Pande, Robert Yu, Florence Manjuh, Edith Welty, Thomas Welty, Laurie Elit, Melissa Lopez-Varon, Jessica Rodriguez, Ellen Baker, Jean-Marie Dangou, Partha Basu, Marie Plante, Fabrice Lecuru, Thomas Randall, Ellen Starr, Joseph Kamgno, Lewis Foxhall, Alan Waxman, Ernest Hawk, Kathleen Schmeler, Sanjay Shete

**Affiliations:** 1 University of Texas MD Anderson Cancer Center, Houston; 2 Cameroon Baptist Convention Health Services, Bamenda, Cameroon; 3 Faculty of Medicine and Biomedical Sciences, University of Yaoundé I, Yaoundé, Cameroon; 4 African Regional Office of the World Health Organization, Brazzaville, Congo; 5 International Agency for Research on Cancer of the World Health Organization, Lyon, France; 6 Division of Gynecologic Oncology, CHU de Quebec, Laval University, Quebec, Canada; 7 Institut Curie, Paris, France; 8 Department of Global Health and Social Medicine, Harvard Medical School, Boston, Massachusetts; 9 Grounds for Health, Williston, Vermont; 10 Department of Obstetrics and Gynecology, University of New Mexico School of Medicine, Albuquerque

## Abstract

**Importance:**

Although Africa has the highest burden of cervical cancer in the world, educational resources to achieve the 90-70-90 targets set by the World Health Organization in its strategy to eliminate cervical cancer are lacking in the region.

**Objectives:**

To adapt, implement, and evaluate the Project Extension for Community Health Care Outcomes (ECHO), an innovative learning tool, to build capacity of clinicians to better incorporate new evidence-based guidelines into cervical cancer control policies and clinical practices.

**Design, Setting, and Participants:**

This cross-sectional study assessed knowledge and practices of clinicians and support staff regarding cervical cancer prevention and control and compared them among respondents who had attended Project ECHO sessions (prior ECHO attendees) with those who had not but were planning on attending in the near future (newcomers) as part of the Cameroon Cervical Cancer Prevention Project ECHO. Satisfaction of prior ECHO attendees was also evaluated. Data were analyzed from January to March 2022.

**Main Outcomes and Measures:**

Main outcomes were practices and knowledge regarding cervical cancer education and prevention and preinvasive management procedures compared among prior ECHO attendees and newcomers.

**Results:**

Of the 75 participants (mean [SD] age, 36.4 [10.0] years; 65.7% [95% CI, 54.3%-77.1%] women) enrolled in this study, 41 (54.7%; 95% CI, 43.1%-66.2%) were prior ECHO attendees, and most were clinicians (55 respondents [78.6%; 95% CI, 68.7%-88.4%]). Overall, 50% (95% CI, 37.8%-62.2%) of respondents reported performing cervical cancer screening with visual inspection of the cervix after application of acetic acid (VIA) and/or visual inspection of the cervix after application of Lugol’s iodine (VILI), 46.3% (95% CI, 34.0%-58.5%) of respondents reported performing human papillomavirus (HPV) testing, and 30.3% (95% CI, 18.9%-41.7%) of respondents reported performing cervical cytological examination in their practices, Approximately one-fourth of respondents reported performing cryotherapy (25.4% [95% CI, 14.7%-36.1%]), thermal ablation (27.3% [95% CI, 16.2%-38.3%]) or loop electrosurgical excisional procedure (LEEP, 25.0% [95% CI, 14.4%-35.6%]) for treatment of preinvasive disease. The clinical use of many of these screening and treatment tools was significantly higher among prior ECHO attendees compared with newcomers (VIA/VILI: 63.2% [95% CI, 47.4%-78.9%] vs 33.3% [95% CI, 16.0%-50.6%]; *P* = .03; cryotherapy: 40.5% [95% CI, 24.3%-56.8%] vs 6.7% [95% CI, 0.0%-15.8%]; *P* = .002; thermal ablation: 43.2% [95% CI, 26.9%-59.6%] vs 6.9% [95% CI, 0.0%-16.4%]; *P* = .002). Knowledge about cervical cancer education, prevention, and management procedures was satisfactory in 36.1% (95% CI, 23.7%-48.5%) of respondents; this proportion was significantly higher among prior ECHO attendees (53.8% [95% CI, 37.7%-69.9%]) compared with newcomers (4.5% [95% CI, 0.0%-13.5%]; *P* < .001). Approximately two-thirds of participants (68.8% [95% CI, 51.8%-85.8%]) reported that they had applied knowledge learned in our ECHO sessions to patient care in their practice or adopted best-practice care through their participation in this ECHO program.

**Conclusions and Relevance:**

These findings suggest that the Project ECHO e-learning and telementoring program was associated with improved skills for clinicians and support staff and enhanced quality of care for patients. In the COVID-19 era and beyond, reinforced efforts to strengthen cervical cancer knowledge and best practices through distance learning and collaboration are needed.

## Key Points

**Question:**

What were the knowledge and practice outcomes associated with participating in an e-learning program to address cervical cancer prevention for health care practitioners in Cameroon?

**Findings:**

In this cross-sectional study of 75 health care practitioners, 53.8% of prior ECHO attendees and 4.5% of newcomers had a satisfactory knowledge score, a significant difference. Approximately two-thirds of participants reported having adopted best practice care or applied knowledge learned through the program to patient care in their practice.

**Meaning:**

These findings suggest that this educational program was associated with improved knowledge and evidence-based practices and higher quality patient care in underresourced areas.

## Teaser

This cross-sectional study assesses a distance learning and telementoring program to build cervical cancer screening and treatment capacity of health care practitioners in Africa.

## Introduction

In its 2020 global strategy to eliminate cervical cancer as a public health problem, the World Health Organization (WHO) set the 90-70-90 targets (90% rate of human papillomavirus [HPV] vaccination, 70% coverage rate of cervical cancer screening, and 90% treatment rate of cervical preinvasive and invasive disease) as a goal that should be achieved by countries across the world by 2030.^1^ With most cases and deaths of this preventable condition occurring in resource-limited settings,^2^ greater efforts are urgently needed to achieve these targets in such settings. Because it lacks most of the resources required to widely implement the 2020 WHO strategy, Africa is the continent with the highest burden of cervical cancer,^3^ and this burden is projected to increase in the coming years if immediate actions are not taken. To date, only a few African countries have effectively introduced the HPV vaccine in their national immunization programs.^4^ Furthermore, screening coverage for cervical cancer and treatment of preinvasive disease in most African countries is reportedly low, especially in rural and underserved communities.^5,6^ This is mainly driven by a glaring shortage of qualified health care workers adequately trained to provide targeted community education and appropriate care in these countries, especially in the field of oncology.^7,8^ Thus, to achieve the ambitious goal to eliminate cervical cancer in high-burden settings like Africa, the WHO recommends engaging and enhancing health care workforce skills and education through the development of training hubs in centers of excellence and the use of telementoring and remote learning to reduce health inequities in underserved communities.^1^ With the rapid spread of the COVID-19 pandemic that resulted in social isolation, distance learning has gained more attention as a valuable tool to spread knowledge, including in Africa where access to new technologies of communication and internet network connectivity is rapidly expanding.^9^

A model of health care education and mentoring, known as *Project ECHO* (Extension for Community Healthcare Outcomes), has been developed to address the inadequate supply of trained personnel in underserved areas.^10^ Established at the University of New Mexico Health Sciences Center, Project ECHO was designed to develop capacity for safe and effective education, prevention, and management of various conditions while monitoring outcomes to ensure quality of care.^10^ The ECHO model is a distance education tool in which specialists, typically located at academic medical centers, connect via teleconferencing technology with community-based practitioners for the purpose of facilitating case-based learning and continuing education. The model has been shown to be effective for teaching community health care practitioners to treat infectious diseases,^11^ and is being applied to many other conditions. For ECHO programs to effectively address common health problems in Africa, they must engage clinicians who have a good understanding of the African health problems and systems and available treatment modalities, so they can provide appropriate recommendations to Africa-based clinicians who have to treat patients with complicated conditions with limited resources. This is of particular concern for cervical cancer because the HIV pandemic, which substantially contributes to the higher burden of cervical cancer while negatively affecting its natural history in terms of HPV acquisition,^12^ persistence,^13^ and occurrence of cervical disease,^14^ disproportionately affects women living in Africa.^15^ In addition, ablative therapies (ie, cryotherapy and thermal ablation) used to treat cervical precancerous lesions in low-resource settings are not recommended in high-income countries. Therefore, cervical cancer experts involved in the training of African health care practitioners should have strong knowledge and experience in using such tools and in treating patients whose clinical presentation may differ from those more commonly observed in high-income countries.^16^

To make the ECHO model more adapted to the African setting and to address various cultural barriers to its implementation, we set up a context-specific and resource-tailored ECHO-modeled learning program focusing on cervical cancer prevention and control through a close consultation of organizations and experts actively involved in cervical cancer control activities in Africa. This study describes the Cameroon Cervical Cancer Prevention Project ECHO and assesses participants’ perceptions of its impact on the performance of clinicians and on their provision of best practice care to their patients.

## Methods

This cross-sectional study was approved by the University of Texas MD Anderson Cancer Center Institutional Review Board. All participants provided written informed consent. We used the Research Electronic Data Capture (REDCap) software (Vanderbilt University)^17^ to administer the survey online. This study followed the Strengthening the Reporting of Observational Studies in Epidemiology (STROBE) reporting guideline for cross-sectional studies.

### Development of the Cameroon Cervical Cancer Prevention Project ECHO

The Cameroon Cervical Cancer Prevention Project ECHO was launched in June 2018 and focused on cervical cancer education, promotion of HPV vaccination, screening and early detection, treatment of preinvasive and early-stage invasive cervical disease, and follow up after treatment. At that time, an established cervical cancer prevention program in West Africa run by the Women’s Health Program (WHP) of the Cameroon Baptist Convention Health Services (CBCHS), a faith-based organization, was seeking to introduce a continuing professional development tool to provide consultation on difficult cases and to regularly update the knowledge and skills of clinicians involved in cervical cancer care services within its wide network of health facilities disseminated over the country.^18^ After signing a Statement of Collaboration with the ECHO Institute in July 2018, a CBCHS associate director attended the ECHO immersion training, and CBCHS became the first ECHO hub in West Africa dedicated to cervical cancer prevention.^18^ This was done in a context where emerging and more effective tools or strategies for screening and treating cervical preinvasive disease were endorsed by international organizations for use in high burden settings^19^ and gradually incorporated in national cancer control policies. Since its inception in 2007, the WHP periodically updates its guidelines for cervical cancer screening and treatment to align them with the most recent WHO recommendations and is staffed by nurses who initially receive 48 hours of training, are mentored by experienced nurses for 1 to 2 months, and must pass a test before practicing independently.^5,20^ New equipment for diagnosing and treating cervical precancers was acquired, and health care practitioners received frequent updated training on cervical cancer screening and prevention strategies, including digital cervicography, thermal ablation, and loop electrosurgical excision procedure (LEEP), with the support of partnering organizations, such as the American Society of Colposcopy and Cervical Pathology.^21^ Cameroon Project ECHO is an economical approach for continuing education of health care practitioners to prevent and treat cervical cancer, combining distance-based learning sessions and expert consultation for management of complicated patients. The program is growing, and health care practitioners from various African countries, including Nigeria, Gambia, Malawi, South Africa, Tanzania, Uganda, Rwanda, Morocco, Ethiopia, and Kenya, attend these monthly sessions.

### Organization and Conduct of a Cameroon Cervical Cancer Prevention Project ECHO Session

The Project ECHO community consists of 3 groups of participants: (1) facilitators and administrative personnel who coordinate the ECHO hub, invite participants to engage in the case-based learning and experts to present a short didactic, handle the teleconferencing equipment, anticipate and address any technical problem encountered during ECHO session, record the ECHO session and share the video recordings with participants, monitor attendance, and help with muting and unmuting participants when necessary; (2) mentors, including clinical specialists or public health experts located in academic or tertiary health centers in Africa, Europe, and North America who lead the case-base discussions and give the didactic lecture; and (3) mentees, including frontline clinicians who select and present clinical cases and other health care practitioners who join the ECHO call to share their experience and learn from their peers. The sessions are interactive, and participants converse in a mutually respectful manner. Although mentors are renowned cervical cancer experts with established competence in the field, the learning process is bidirectional, as mentors can equally learn from mentees who share field experience in their local community. Thus, every participant learns during these sessions, one of the key tenets of the ECHO concept.

Participation in our ECHO sessions is free but requires attendees to have an electronic device (smartphone, tablet, or computer) connected to the internet. To join an ECHO session, participants are provided with instructions for how to virtually connect to the videoconference using Zoom software (Zoom Video Communications).^22^ These Project ECHO sessions are held monthly and last between 60 and 90 minutes.

### Evaluation of the ECHO Program

#### Survey Design

In 2021, we developed a comprehensive survey to assess the training, knowledge, and practices with regard to cervical cancer control among clinicians, support staff, and other health care practitioners invited to participate in our ECHO sessions. We ensured that the survey is consistent with the levels of evidence described in the Moore’s evaluation framework^23^ (eTable 1 in the Supplement). Moore’s framework is used to assess current evidence from Project ECHO programs because it focuses on the target outcomes of Continuing Medical Education (CME) events with the goal of iteratively modifying the design of the event to achieve the intended results. The final version of the survey was tested and validated by international experts and Cameroon and other African health care practitioners.

#### Survey Content

We collected sociodemographic information of respondents, including age, sex, type of practitioner (clinicians or nonclinicians [eg, health administrators, community workers, program managers, pharmacists, and laboratory technicians], and facility where they work [ie, primary, secondary, tertiary, or research]). We assessed their knowledge with 24 questions about cervical cancer risk factors, HPV vaccination, cervical cancer screening, colposcopy, treatment, and follow-up of preinvasive disease. We asked Project ECHO attendees about the organization of and their satisfaction with the ECHO sessions and how participation in these ECHO sessions impacted their self-efficacy and their quality of care. Completing the survey took approximately 15 minutes. Respondents could skip any question at their discretion.

#### Survey Administration

After institutional review board approval, we invited eligible health practitioners who were notified of our ECHO sessions to participate in the survey. Participation was anonymous and voluntary. No compensation or incentive was provided, and refusal to take the survey had no consequences on future participation in ECHO sessions. In the invitation letter that was sent to participants by email, the purpose of the survey was further explained, and a link was provided to access the survey. The survey was administered between May and July 2021. We sent invited participants a maximum of 2 reminder emails (at 1-week intervals) after receiving the initial invitational email. Of 300 people who received the survey, 77 (25.7%) consented to complete it. Two new enrollees who reported that they were not planning on attending our ECHO program in the near future were not eligible to participate in this study.

### Statistical Analysis

To better describe our study population and their perceptions of the impact of this program, we classified participants into 2 groups according to their ECHO attendance: respondents who had ever attended Project ECHO sessions at the time of the survey (prior ECHO attendees) and those who were new to the program and had not attended Project ECHO sessions yet but were planning on attending in the near future (hereafter, *newcomers*). In each group, we calculated descriptive statistics. Comparisons between groups were made with the Fisher Exact test for categorical variables and the *t* test for continuous variables. Two-sided *P* < .05 was considered statistically significant. The knowledge score was developed by assigning +1 point to each correct answer, −1 point to each incorrect answer, and 0 to each do not know answer. For a given respondent, the total knowledge score was obtained by summing scores from all 24 knowledge questions. A priory, a knowledge score of 12 points or higher was considered satisfactory, while a knowledge score of 11 points or less was considered unsatisfactory. Data were analyzed from January to March 2022.

## Results

A total of 75 eligible participants (mean [SD] age, 36.4 [10.0] years; 46 [65.7%; 95% CI, 54.3%-77.1%] women) completed the survey, with most respondents being clinicians (55 respondents [78.6%; 95% CI, 68.7%-88.4%]) (Table 1). A total of 41 respondents (54.7%; 95% CI, 43.1%-66.2%) were prior ECHO attendees, and 34 respondents (45.3%; 95% CI, 33.8%-56.9%) were newcomers. At the time of the survey, prior ECHO attendees had attended a mean (SD) of 12.3 (10.2) ECHO sessions. Participants were from 13 African countries (Burundi, Cameroon, Cote d’Ivoire, Democratic Republic of Congo, Ethiopia, Gabon, Kenya, Morocco, Nigeria, Senegal, South Africa, Chad, and Uganda).

**Table 1.** Sociodemographic Characteristics of the Study Sample

	Total (N = 75)^a^		Prior ECHO attendees (n = 41)		Newcomers (n = 34)^b^		*P* value^c^
	No.	% (95% CI)	No.	% (95% CI)	No.	% (95% CI)	
Age, mean (SD)	36.4 (10.0)	NA	38.6 (10.3)	NA	33.6 (9.0)	NA	.48
Sex							
Men	24	34.3 (22.9-45.7)	14	35.0 (19.8-50.2)	10	33.3 (16.0-50.6)	>.99
Women	46	65.7 (54.3-77.1)	26	65.0 (49.8-80.2)	20	66.7 (49.4-84.0)	
Practitioner type							
Clinicians	55	78.6 (68.7-88.4)	34	85.0 (73.7-93.3)	21	70.0 (53.2-86.8)	.15
Nonclinicians^d^	15	21.4 (11.6-31.3)	6	15.0 (3.7-26.3)	9	30.0 (13.2-46.8)	
Facility							
Primary	19	27.1 (16.5-37.8)	9	22.5 (9.2-35.8)	10	33.3 (16.0-50.6)	.33
Secondary	14	20.0 (10.4-29.6)	8	20.0 (7.3-32.7)	6	20.0 (5.3-34.7)	
Tertiary	23	32.9 (21.6-44.1)	12	30.0 (15.4-44.6)	11	36.7 (19.0-54.3)	
Other^e^	14	20.0 (10.4-29.6)	11	27.5 (13.3-41.7)	3	10.0 (0.0-21.0)	

Abbreviation: ECHO, Extension for Community Healthcare Outcomes.

^a^ The respondents who provided incomplete or no information about their sociodemographics were not included in this table. For this reason, the total number of respondents may not add up to 75.

^b^ Newcomers are participants who have never attended ECHO sessions, but were eligible to take the survey because they indicated that they were willing to participate in these ECHO sessions in the next 6 months.

^c^ The *P* value was calculated using the *t* test for continuous variables and the Fisher exact test for categorical variables.

^d^ The nonclinicians category included health administrators, community workers, program managers, pharmacists, and laboratory technicians.

^e^ The other category included research institutions, ministries of health, and other nonclinical settings.

### ECHO Attendance

At the program’s inception in June 2018, the number of participants (mainly from Cameroon, where the Project ECHO hub is located) varied between 10 and 15 per monthly ECHO session; this number gradually increased with the number of participating countries to reach a mean of approximately 35 to 40 participants per monthly session. As of August 2022, a total of 50 ECHO sessions had been held, with 98 clinical cases presented and 47 didactic lectures given.

### Practice of Cervical Cancer Prevention and Treatment Techniques

Participants were surveyed about their current practice of cervical cancer prevention and treatment techniques (eTable 2 in the Supplement). Overall, half of respondents (50% [95% CI, 37.8%-62.2%]) reported performing visual inspection with acetic acid (VIA) or visual inspection with Lugol’s iodine (VILI) in their clinical practice. This proportion was 2 times higher among prior ECHO attendees compared with newcomers (63.2% [95% CI, 47.4%-78.9%] vs 33.3% [95% CI, 16.0%-50.6%]; *P* = .03). Less than half of respondents reported using cervical cytological examination (30.3% [95% CI, 18.9%-41.7%]) or HPV testing (46.3% [95% CI, 34.0%-58.5%]) for cervical cancer screening in their practice.

Approximately one-fourth of respondents reported performing cryotherapy (25.4% [95% CI, 14.7%-36.1%]), thermal ablation (27.3% [95% CI, 16.2%-38.3%]), and LEEP (25.0% [95% CI, 14.4%-35.6%]) in their clinical practice. The proportion of respondents who were performing ablative therapy procedures was significantly higher among prior ECHO attendees compared with newcomers (cryotherapy: 40.5% [95% CI, 24.3%-56.8%] vs 6.7% [95% CI, 0.0%-15.8%]; *P* = .002; thermal ablation: 43.2% [95% CI, 26.9%-59.6%] vs 6.9% [95% CI, 0.0%-16.4%]; *P* = .002) (eTable 2 in the Supplement).

### Knowledge About Cervical Cancer Education, Screening, and Preinvasive Disease Management Procedures

Among 61 participants who responded to the knowledge questions, the knowledge score ranged from −1 to 18 points. Overall, 36.1% (95% CI, 23.7%-48.5%) of respondents had a satisfactory knowledge score (≥12 points). The distribution of the knowledge score differed between prior ECHO attendees and newcomers (Figure). Specifically, the proportion of respondents who achieved a satisfactory knowledge score was significantly higher among prior ECHO sessions attendees compared with newcomers (58.3% [95% CI, 37.7%-69.9%] vs 4.5% [95% CI, 0.0%-13.5%]; *P* < .001).

**Figure.**
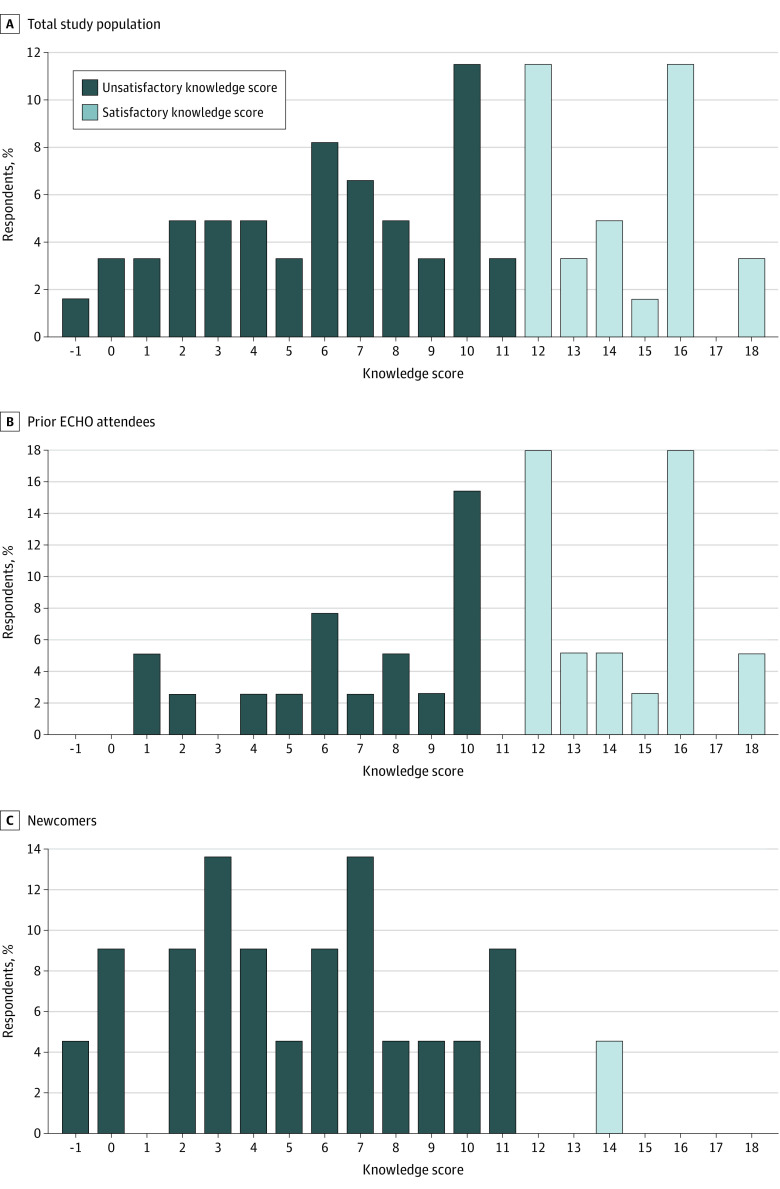
Distribution of the Knowledge Score Overall, and According to ECHO (Extension for Community Health Care Outcomes) Attendance Scores were dichotomized as unsatisfactory (≤11) or satisfactory (≥12).

### Evaluation of Project ECHO

#### Satisfaction

All Project ECHO attendees who responded stated they enjoyed the opportunity to learn with peers, and 96.9% (95% CI, 90.5%-100.0%) of attendees considered that the opportunity to connect with academic specialists was important to them (Table 2). Up to 66.7% (95% CI, 48.8%-84.6%) of ECHO attendees reported being confident when presenting patient cases during our ECHO sessions. Most attendees reported that the topics presented in didactics were relevant to their practice (96.6% [95% CI, 90.5%-100.0%]) and that the level of detail in the didactics was appropriate (84.4% [95% CI, 71.1%-97.7%]). Approximately two-thirds of respondents (62.5% [95% CI, 44.8%-80.2%]) indicated that there was enough time for questions and discussion during the ECHO sessions, while nearly all attendees (96.9% [95% CI, 90.5%-100.0%]) reported that they learned from community practitioner peers who presented their patient cases during these ECHO sessions.

**Table 2.** Satisfaction of the Cervical Cancer Prevention Project ECHO Attendees

Evaluation statements^a^	Agree		Neutral		Disagree	
	No.	% (95% CI)	No.	% (95% CI)	No.	% (95% CI)
I enjoy the opportunity to learn with peers	32	100.0	0	0.0	0	0.0
The opportunity to connect with academic specialists is important to me	31	96.9 (90.5-100.0)	1	3.1 (0.0-9.5)	0	0.0 (0.0-0.0)
I am confident when presenting patient cases during ECHO sessions	20	66.7 (48.8-84.6)	9	30.0 (12.6-47.4)	1	3.3 (0.0-10.2)
The topics presented in didactics are relevant to my practice	31	96.9 (90.5-100.0)	1	3.1 (0.0-9.5)	0	0.0 (0.0-0.0)
The level of detail in the didactics is appropriate	27	84.4 (71.1-97.7)	4	12.5 (0.4-24.6)	1	3.1 (0.0-9.5)
There is enough time for questions and discussion during the ECHO sessions	20	62.5 (44.8-80.2)	8	25.0 (9.1-40.9)	4	12.5 (0.4-24.6)
I learn from providers who present their patient cases during ECHO sessions	31	96.9 (90.5-100.0)	1	3.1 (0.0-9.5)	0	0.0 (0.0-0.0)

Abbreviation: ECHO, Extension for Community Healthcare Outcomes.

^a^ For certain variables or statements, the total number of observations is not equal to 32 because of missing data.

#### Opportunities to Improve Participation

All Project ECHO attendees who responded rated the organization and the conduct of these ECHO sessions as good or excellent (Table 3). Most respondents reported that the opportunity to earn continuing education credits through these ECHO sessions was important to them (84.4% [95% CI, 71.1%-97.7%]), and that they would be more likely to attend these ECHO sessions if they received a certificate of attendance (87.5% [95% CI, 75.4%-99.6%]). However, more than half of the respondents (56.3% [95% CI, 38.1%-74.4%]) indicated that they had experienced technical problems connecting to an ECHO session and/or keeping a connection at least once.

**Table 3.** Opportunities to Improve Participation in the Cervical Cancer Prevention Project ECHO

Evaluation statements^a^	No.	% (95% CI)
How would you rate the organization and conduct of the ECHO sessions?		
Excellent	15	46.9 (28.6-65.2)
Good	17	53.1 (34.8-71.4)
Fair	0	0
Average/poor	0	0
How important is the opportunity to earn continuing education (CNE/CME) credits through the ECHO sessions?		
Important	27	84.4 (71.1-97.7)
Neutral	5	15.6 (2.3-28.9)
Not important	0	0
How important is the opportunity to receive a summary of recommendations from the faculty after the ECHO sessions?		
Important	32	100.0
Neutral	0	0
Not important	0	0
Would you be more likely to attend ECHO sessions, if you received a certificate of attendance?		
Yes	28	87.5 (75.4-99.6)
No	2	6.3 (0.0-15.1)
Don’t know	2	6.3 (0.0-15.1)
Would you be more likely to prepare and present a case, if you received a certificate for presenting it?		
Yes	25	80.6 (65.9-95.4)
No	4	12.9 (0.4-25.4)
Don’t know	2	6.5 (0.0-15.6)
During the last ECHO session you attended, have you experienced technical problems connecting to the ECHO meeting and/or keeping a connection?		
Yes	18	56.3 (38.1-74.4)
No	14	43.8 (25.6-61.9)

Abbreviation: ECHO, Extension for Community Healthcare Outcomes.

^a^ For certain variables or statements, the total number of observations is not equal to 32 because of missing data.

#### Perceived Impact of ECHO Sessions on Attendees’ Knowledge, Skills, and Practice and on Patient Care

Most attendees reported that participation in the Project ECHO program had enhanced their ability to obtain specialty consultations (80.6% [95% CI, 65.9%-95.4%]), had improved the coordination of care for their patients (83.9% [95% CI, 70.2%-97.6%]), had increased their ability to offer more comprehensive care to their patients (93.8% [95% CI, 84.9%-100.0%]), and was an effective way to enhance their expertise (93.5% [95% CI, 84.4%-100.0%]) (Table 4). Most respondents reported that they had applied knowledge learned in ECHO sessions to patient care in their practice (68.8% [95% CI, 51.8%-85.8%]), that they had adopted best-practice care through their participation in our ECHO program (68.8% [95% CI, 51.8%-85.8%]), that they had developed additional clinical expertise through ECHO sessions (78.1% [95% CI, 63.0%-93.3%]), and that they were interested in continuing participation in ECHO sessions to improve their knowledge and skills (93.8% [95% CI, 84.9%-100.0%]).

**Table 4.** Perceived Impact of the Cervical Cancer Prevention Project ECHO on the Knowledge, Skills, and Practice of ECHO Attendees

Evaluation statements^a^	Agree		Neutral		Disagree	
	No.	% (95% CI)	No.	% (95% CI)	No.	% (95% CI)
Participation in these Cervical Cancer Prevention ECHO sessions has enhanced my ability to obtain specialty consultations.	25	80.6 (65.9-95.4)	5	16.1 (2.4-29.8)	1	3.2 (0.0-9.8)
Consultations through ECHO sessions save me time.	16	51.6 (33.0-70.2)	13	41.9 (23.5-60.3)	2	6.5 (0.0-15.6)
ECHO session participation improved the coordination of care for my patients.	26	83.9 (70.2-97.6)	5	16.1 (2.4-29.8)	0	0.0 (0.0-0.0)
ECHO sessions have enhanced the quality of life for my patients.	21	70.0 (52.6-87.4)	1	3.3 (0.0-10.2)	8	26.7 (9.9-43.5)
I enjoy consulting with specialists about my patients’ management	26	83.9 (70.2-97.6)	4	12.9 (0.4-25.4)	1	3.2 (0.0-9.8)
I have applied knowledge learned in ECHO sessions to patients in my practice.	22	68.8 (51.8-85.8)	8	25.0 (9.1-40.9)	2	6.3 (0.0-15.1)
I have adopted best practice care through my participation in ECHO sessions.	22	68.8 (51.8-85.8)	8	25.0 (9.1-40.9)	2	6.3 (0.0-15.1)
I have developed additional clinical expertise through ECHO sessions.	25	78.1 (63.0-93.3)	5	15.6 (2.3-28.9)	2	6.3 (0.0-15.1)
I am interested in continuing participation in ECHO sessions to improve my knowledge and skills.	30	93.8 (84.9-100.0)	2	6.3 (0.0-15.1)	0	0
Participating in ECHO sessions is an effective way for my clinic to enhance its expertise.	29	93.5 (84.4-100.0)	2	6.5 (0.0-15.6)	0	0
Participation in ECHO sessions increases my ability to offer more comprehensive care.	30	93.8 (84.9-100.0)	2	6.3 (0.0-15.1)	0	0
Administration and staff at my practice are supportive of my involvement in Project ECHO.	25	80.6 (65.9-95.4)	6	19.4 (4.6-34.1)	0	0

Abbreviation: ECHO, Extension for Community Healthcare Outcomes.

^a^ For certain variables and statements, the total number of observations is not equal to 32 because of missing data.

## Discussion

In this cross-sectional study, we assessed an innovative and culturally appropriate distance learning ECHO-modeled program that engages international and African cervical cancer experts and fosters collaborations between Africa-based institutions to spread knowledge and facilitate experience-sharing among African health care practitioners involved in cervical cancer prevention and control activities, without having to move patients or practitioners from their communities. This resource-tailored program was associated with improved competencies and evidence-based practices for clinicians, their support staff, and other health care practitioners, who reported improved outcomes for their patients. These findings suggest that this program has the potential to reduce health inequities in cervical cancer control in the region. Overall, our ECHO attendees reported being satisfied with the e-learning program.

In Africa, community health care practitioners are at the forefront of caring for women at risk for or diagnosed with cervical cancer and are often the point of referral to specialist consultation.^24^ Our study suggests an opportunity to further enhance the knowledge and practices of community-based health care practitioners on cervical cancer prevention and care services. In our study, level of knowledge about cervical cancer among participating African health care practitioners was low, with just a few respondents who had not attended our ECHO sessions at the time of survey (newcomers) having a satisfactory knowledge score, compared with more than half of the health care practitioners who had attended our ECHO program (prior ECHO attendees). This stresses the need for policy makers to widely promote the development and use of effective educational interventions for health care practitioners, including formal training of nurses on cervical cancer prevention and treatment, so that they can effectively use ECHO sessions to rapidly improve knowledge and practices and to provide better care for their patients. A lower rate of health care practitioners newly enrolled in our ECHO program (newcomers) reported performing VIA/VILI, cryotherapy, or thermal ablation compared with prior ECHO attendees. Thermal ablation is a recently recommended tool for the treatment of cervical preinvasive lesions in limited resource settings and has the potential to link cervical cancer screening with treatment of precancerous lesions in a single visit or just a few visits (ie, a see-and-treat approach).^25^

An important aspect of our ECHO program was to build up a virtual community of practice.^26^ Reports about communities of practice demonstrate the critical role they play in learning and collaboration by providing opportunities to overcome challenges related to in-person interaction, such as linking geographically dispersed networks of people with common interests.^27,28^ This is even more needed in the COVID-19 era and consequential measures restricting movements and social interactions. Our ECHO program created an interactive platform in which experts and specialists from renowned organizations in Africa, Europe, and North America could connect on a regular basis with health care practitioners involved in cervical cancer control activities in Cameroon and other African countries. Active participation and engagement are instrumental to ensure the sustainability of virtual communities of practice, like Project ECHO-built communities. Active participation is fostered by having supportive leadership, including local champions, and active facilitation.^27,28^ In our experience, the involvement of local champions, like the CBCHS leaders, was essential to boost participation and engagement from health care practitioners in the Project ECHO program. Regional, onsite clinical mentors encouraged participation in our program, assisted with participant case-presentation development, and often continued discussions with participants after sessions concluded. The degree to which an innovative approach or intervention can be incorporated into existing organizations is an important factor in its sustainable implementation.^29^

To date, participants in the Project ECHO program do not receive a certificate for attending the sessions. Our findings that the health care practitioners would be more likely to participate in these ECHO sessions if certificates of attendance or CME credits were issued suggests that the provision of such credits or certificates may serve as an important motivating factor, improving active participation and retention. To address this need, we are working with partnering institutions to provide certification to participants in these sessions. It is necessary to raise awareness of the policies and regulations related to CME for health care practitioners in African countries that require these credits for the practitioners to maintain their license, thereby creating an incentive for individuals to take part in our ECHO sessions.

A major challenge posed by participants in our ECHO sessions was related to the poor quality of internet connection. More than half of participants reported experiencing technical problems connecting to and/or keeping a connection with our ECHO sessions. This technical issue, which reflects the inadequate level of information and telecommunication infrastructure in Africa, adversely affected active participation of some health care practitioners in these ECHO sessions. For participants who were not able to follow the live sessions, video recordings were provided at the end of each session and could be watched at any time when their internet connection became more stable. Moreover, a written summary of recommendations recapping the case-based discussions was also provided to participants, which they could keep and use later as needed.

The higher knowledge among prior ECHO attendees compared with the newcomers found in this study cannot be entirely attributed to the ECHO attendance, as many of the ECHO attendees had additional training. Indeed, participants from WHP (a subset of ECHO attendees) were well equipped and trained at performing cervical screening and preinvasive treatment procedures outside ECHO.^20^ However, even for this group of participants, ECHO provides continued mentorship to improve patient care for complicated or equivocal situations encountered in clinical practice. This is reflected by the fact that participants self-reported improved knowledge and enhanced clinical expertise gained in attending ECHO sessions, including in the presence of prior training, highlighting the value of Project ECHO. While ECHO does not replace basic and hands-on training, it serves as a practical and complementary tool to enhance knowledge sharing among health care practitioners and extend the provision of evidence-based practices through continuing education. For participants who are less skilled, ECHO is intended to provide an opportunity to learn not only from their peers who are more experienced, but also from renowned experts who have established competence in the field, thereby contributing to reduction in the knowledge gap among health care practitioners.

### Limitations

This study was subject to limitations. First, while the evaluation demonstrated a higher level of knowledge and self-efficacy among prior ECHO attendees compared with newcomers, our study did not directly assess the impact of the ECHO sessions on patient outcomes, since cervical precancer and cancer are slowly progressing conditions. However, most ECHO attendees reported that they had applied knowledge learned in ECHO sessions to their patients and that enhanced the quality of life of their patients, the ultimate beneficiaries of the program. Second, since health care practitioners on the ECHO mailing list were invited to participate in the survey on a voluntary basis with no incentive, only one-fourth (25.7%) of invited participants completed the survey. Third, the difference in knowledge observed between ECHO attendees and newcomers could be partly explained by other factors that could not be controlled in this cross-sectional analysis, such as educational background, training from other sources, or potential differences in study enrollment through the study period. Fourth, we could not quantify the changes in participants’ individual knowledge or their level of satisfaction with the program over time, as the cross-sectional data used in this study referenced only the first round of the survey we administered as part of this program. However, we plan to administer the survey at additional time points (ie, at 6-month or 12-month intervals) going forward in order to measure the longitudinal variation in these metrics.

## Conclusions

The findings of this cross-sectional study suggest that the Project ECHO program provided a robust model for addressing cervical cancer in underresourced areas of Africa through utilization of technology-enabled collaborative learning and support for frontline health care practitioners. While this e-learning program could contribute to improved health care practitioners’ knowledge and skills and improved best practice care for patients, its sustainability depends on a number of factors, including contextual adaption and adoption by African-based institutions. In the COVID-19 era and beyond, such telementoring programs may offer significant practical advantages. Such programs allow for social distancing and reduce movements of clinicians and patients while enabling them to receive better care in their community.

## Notes

Supplement. **eTable 1.** Outcome of the Cameroon Cervical Cancer Prevention ECHO Program in Relation to Moore’s Levels of Expanded Framework **eTable 2.** Assessment of Practices of Cervical Cancer Prevention and Management Techniques by ECHO Attendees and Newcomers
