# Supplementary material for: Development, Implementation, and Evaluation of a Distance Learning and Telementoring Program for Cervical Cancer Prevention in Cameroon
Source: JAMA Netw Open. 2022 Nov 8;5(11):e2240801. doi: 10.1001/jamanetworkopen.2022.40801 (PMC9644259; doi:10.1001/jamanetworkopen.2022.40801)
Supplement: Supplement. — eTable 1. Outcome of the Cameroon Cervical Cancer Prevention ECHO Program in Relation to Moore’s Levels of Expanded Framework eTable 2. Assessment of Practices of Cervical Cancer Prevention and Management Techniques by ECHO Attendees and Newcomers [file jamanetwopen-e2240801-s001.pdf]

Supplemental Online Content

Fokom Domgue J, Pande M, Yu R, et al. Development, implementation, and evaluation of a distance learning and telementoring program for cervical cancer prevention in Cameroon. *JAMA Netw Open*. 2022;5(11):e2240801. doi:10.1001/jamanetworkopen.2022.40801

eTable 1. Outcome of the Cameroon Cervical Cancer Prevention ECHO Program in Relation to Moore's Levels of Expanded Framework

eTable 2. Assessment of Practices of Cervical Cancer Prevention and Management Techniques by ECHO Attendees and Newcomers

This supplemental material has been provided by the authors to give readers additional information about their work.

© 2022 Fokom Domgue J et al. *JAMA Network Open.*

eTable 1. Outcome of the Cameroon Cervical Cancer Prevention ECHO Program in Relation to Moore's Levels of Expanded Framework

Levels of expanded framework	Description of level	Outcome evaluation
**Level 1: participation**	Number of health professionals who participated in each ECHO session	Attendance at these ECHO sessions is recorded and well kept.<br>At the beginning of each ECHO session, participants are asked to introduce themselves briefly either orally, or by typing their names and affiliation in the chat window of the zoom page.
**Level 2: satisfaction**	Degree to which expectations of participants about CME activity were met	A survey was completed by providers invited to participate in these cervical cancer prevention ECHO sessions, who had either attended at least one ECHO session (prior ECHO attendees) or were planning on attending our ECHO sessions in the next six months (newcomers). The survey was conducted via an online questionnaire to evaluate the levels of satisfaction of participants with acquired knowledge, class organization, connectivity during the course. Responses options included "disagree", "neither agree nor disagree", and "agree"

© 2022 Fokom Domgue J et al. *JAMA Network Open.*

Levels of expanded framework	Description of level	Outcome evaluation
**Level 3: Learning: knowledge**	Participant statements about what the CME intended them to know	Knowledge questions included 24 questions, to evaluate participants level of knowledge, and how well they change their knowledge about cervical cancer prevention through process of education via attendance at these Cervical Cancer Prevention ECHO sessions
**Level 4: Learning: competence**	The degree to which participants show in an educational setting how to do what the CME activity intended them to be able to do	During each ECHO session, providers presented patient cases selected from their facility (an average of two cases per session). They identified the questions they wanted the specialists/experts and peers to consider about the patient in their presentation in the case-based discussions. After the case-based discussion, a summary of recommendations from the specialists and peers was outlined and shared with participants.<br>The participants also provided recommendations and guidance to their peers during ECHOs session in response to a peer's patient case or during the discussion that followed the case presentation.<br>Survey about competence and professional satisfaction was administered via an online questionnaire.

© 2022 Fokom Domgue J et al. *JAMA Network Open.*

eTable 2. Assessment of Practices of Cervical Cancer Prevention and Management Techniques by ECHO Attendees and Newcomers

Technique/Procedure	Total[a]	Prior ECHO attendees		Newcomers[b]		p-value[c]
	N (%, 95%CI)	n	% (95%CI)	n	% (95%CI)	
**Education**						
**HPV vaccine recommendation**						0.515
**Yes**	55 (83.3 (74.1-92.6))	32	86.5 (75.2 – 97.8)	23	79.3 (64.2 – 94.4)	
**No**	11 (16.7 (7.4 – 25.9))	5	13.5 (2.2 – 24.8)	6	20.7 (5.6 – 35.8)	
**Screening**						
**VIA/VILI**						0.027
**Yes**	34 (50.0 (37.8 – 62.2))	24	63.2 (47.4 – 78.9)	10	33.3 (16.0 – 50.6)	
**No**	34 (50.0 (37.8 – 62.2))	14	36.8 (21.1 – 52.6)	20	66.7 (49.4 – 84.0)	
**Cervical cytology (Pap test)**						0.594
**Yes**	20 (30.3 (18.9 – 41.7))	10	27.0 (12.3 – 41.7)	10	34.5 (16.7 – 52.2)	
**No**	46 (69.7 (58.3 – 81.1))	27	73.0 (58.3 – 87.7)	19	65.5 (47.8 – 83.3)	
**HPV testing**						0.999
**Yes**	31 (46.3 (34.0 – 58.5))	17	45.9 (29.5 – 62.4)	14	46.7 (28.3 – 65.0)	
**No**	36 (53.7 (41.5 – 66.0))	20	54.1 (37.6 – 70.5)	16	53.3 (35.0 – 71.7)	
**Digital Cervicography**						<0.001
**Yes**	18 (26.5 (15.7 – 37.2))	16	42.1 (26.0 – 58.2)	2	6.7 (0.0 – 15.8)	
**No**	50 (73.5 (62.8 – 84.3))	22	57.9 (41.8 – 74.0)	28	93.3 (84.2 – 100.0)	
**Colposcopy, standard**						0.999
**Yes**	14 (20.6 (10.7 – 30.4))	8	21.1 (7.8 – 34.4)	6	20.0 (5.3 – 34.7)	
**No**	54 (79.4 (69.6 – 89.3))	30	78.9 (65.6 – 92.2)	24	80.0 (65.3 – 94.7)	
**Colposcopy, portable**						0.007
**Yes**	8 (11.8 (3.9 – 19.6))	8	21.1 (7.8 – 34.4)	0	0.0 (0.0 – 0.0)	
**No**	60 (88.2 (80.4 – 96.1))	30	78.9 (65.6 – 92.2)	30	100.0 (100.0 – 100.0)	
**Cervical biopsy**						0.029
**Yes**	32 (48.5 (36.1 – 60.9))	22	61.1 (44.8 – 77.5)	10	33.3 (16.0 – 50.7)	
**No**	34 (51.5 (39.1 – 63.9))	14	38.9 (22.5 – 55.2)	20	66.7 (49.3 – 84.0)	

© 2022 Fokom Domgue J et al. *JAMA Network Open.*

**<u>Treatment</u>**						
**Cryotherapy**						0.002
**Yes**	17 (25.4 (14.7 – 36.1))	15	40.5 (24.3 – 56.8)	2	6.7 (0.0 – 15.8)	
**No**	50 (74.6 (63.9 – 85.3))	22	59.5 (43.2 – 75.7)	28	93.3 (84.2 – 100.0)	
**Thermal ablation**						0.002
**Yes**	18 (27.3 (16.2 – 38.3))	16	43.2 (26.9 – 59.6)	2	6.9 (0.0 – 16.4)	
**No**	48 (72.7 (61.7 – 83.8))	21	56.8 (40.4 – 73.1)	27	93.1 (83.6 – 100.0)	
**LEEP/LLETZ**						0.574
**Yes**	17 (25.0 (14.4 – 35.6))	11	28.9 (14.2 – 43.7)	6	20.0 (5.3 – 34.7)	
**No**	51 (75.0 (64.4 – 85.6))	27	71.1 (56.3 – 85.8)	24	80.0 (65.3 – 94.7)	

[a] For certain variables. the total number of observations does not add up to 75, because of missing data.

[b]Newcomers are participants who have never attended our ECHO sessions, but were eligible to take the survey because they indicated that they were willing to participate in these ECHO sessions in the next six months

[c]p-value was calculated using the t-test for continuous variables and the Fisher exact test for categorical variables

VIA: visual inspection of the cervix after application of acetic acid; VILI: visual inspection of the cervix after application of Lugol's iodine; HPV: human papillomavirus; LEEP: Loop electrosurgical excision procedure; LLETZ: large loop excision of the transformation zone; CCS: cervical cancer screening

© 2022 Fokom Domgue J et al. *JAMA Network Open.*
